# Intrinsic and extrinsic motives of undergraduate students for pursuing a master’s degree: Applying the Eccles et al. expectancy-value model

**DOI:** 10.1371/journal.pone.0317204

**Published:** 2025-03-25

**Authors:** Susanne Bergann, Irmela Blüthmann, Martin Neugebauer, Rainer Watermann

**Affiliations:** 1 Unit for Teaching and Education Quality, Freie Universität Berlin, Berlin, Germany; 2 Karlsruhe University of Education, Karlsruhe, Germany; 3 Division of Empirical Research in Education, Freie Universität Berlin, Berlin, Germany; RMIT University, VIET NAM

## Abstract

This study investigates the factors driving students to pursue a master’s degree, taking into account the mediating role of ability beliefs in this decision-making process. Previous research has primarily focused on a narrow range of explanatory factors, such as the utility value of a master’s degree and associated monetary costs. Yet, the role of intrinsic value, expectations of success, and psychological costs of failure, which are crucial in the decision to pursue further studies, have been insufficiently explored. Drawing on the expectancy-value model by Eccles et al., this study develops a comprehensive model to examine the factors influencing bachelor’s students’ intentions to pursue a master’s degree. Using structural equation modelling (SEM) to analyse cross-sectional data from N =  3,044 undergraduate students at a major German university, our findings provide robust support for the theoretical framework. Specifically, students’ expectations of successfully completing a master’s degree emerged as the strongest predictor of their intentions to transition (β = .42, p <  0.001). Intrinsic value, indicated by interest in scientific work, proved to be as important as the utility value (β = .36, p <  0.001). Moreover, psychological costs significantly influenced student’s decisions (β =  -.21, p <  0.01). Notably, apart from academic performance, beliefs about general and scientific abilities contributed to both the expectancy and value components of the model. These results provide valuable insights for higher education institutions regarding programme development and counseling services aimed at supporting students during this critical decision-making process. Additionally, this study enriches the theoretical understanding of the complex dynamics involved in student’s academic transitions.

## Introduction

Although the distinction between bachelor’s and master’s degrees in higher education has a long tradition in the United States, this distinction is a relatively recent development in most European countries, including Germany, having been introduced in the past 25 years as part of the Bologna reforms. These reforms aimed to create standardised degrees across Europe to facilitate student mobility [1]. As a first professional qualification, the bachelor’s degree has become the standard degree awarded by European higher education institutions (HEIs), typically leading to entry-level jobs [1]. After completing their bachelor’s degree, academically qualified graduates have the opportunity to continue on to a master’s degree, which qualifies them for a doctoral programme and is a prerequisite for careers in academia and research.

The decision-making period for undergraduate students represents a transitional phase. Compared to other educational transitions - such as from primary to secondary education or from secondary school to university - the transition from undergraduate to postgraduate studies has received limited attention in previous research [2–5].

Key questions arise: Who decides to pursue a master’s degree, when do students make this decision, and why? Addressing these questions is important for several reasons. First, it is essential for programme developers at HEIs to know how many students plan to pursue a master’s degree, when they decide to do so, and what their motivations are (e.g., [6]). Second, HEIs can use this information to design master’s programmes that align more closely with the interests and aspirations of prospective students. Third, HEIs can work to reduce informal barriers to entry and help students to make informed choices about their educational careers. In summary, enhancing our understanding of the factors influencing the choice of master’s degree programmes can facilitate informed decision-making for both students and HEIs.

Furthermore, understanding why students pursue master’s degrees is particularly relevant in the light of high transition rates observed in European universities, even two decades after the Bologna reforms [5,7,8]. It is possible that students feel compelled to obtain a master’s degree in order to improve their career prospects. However, intrinsic motivations, such as a genuine interest in scientific work, may also play a role.

Previous research on the transition from undergraduate to postgraduate studies has mainly focused on analysing social inequalities, including gender and ethnic disparities [3–5,8–12]. These studies mainly use sociological rational choice models [13,14], which attribute social disparities in educational choices to differences in anticipated monetary returns, perceived probabilities of success, and financial costs associated with different educational pathways. However, little is known about the impact of intrinsic motives (e.g., interest in scientific work) or psychological costs (e.g., concern about disappointing parental educational aspirations) in choosing a master’s programme. To our knowledge, a comprehensive study, which considers these decision-making factors and investigates them independently of the issue of social disparities, is lacking. Given the practical implications outlined, it seems necessary to extend the theories proposed in previous research and to use a broader theoretical framework.

This study addresses this research gap by theoretically discussing and empirically testing the applicability of Eccles and colleagues’ psychological expectancy-value model [15–18] in the context of the transition from undergraduate to postgraduate studies. The Eccles et al model extends sociological rational choice models by incorporating additional value components, such as intrinsic motivations and psychological costs, as well as determinants of expectancy and value components. thereby contributing to a better understanding of the transition. While the expectancy-value model has gained significant attention and acclaim, its applicability to the undergraduate-to-postgraduate transition remains largely unexplored in the existing literature.

We will first discuss how the Eccles et al. model can help us understand this transition and formulate corresponding hypotheses. Then we will present empirical analyses based on a cross-sectional dataset of *N* =  3,044 undergraduate students enrolled at a major German university. Germany serves as a test case because it has the largest number of university students in Europe [19]. The combination of low tuition fees and the relatively recent implementation of the bachelor-master transition as part of the Bologna process positions Germany as a prototype for many European countries, making our findings particularly relevant. In the conclusion of this paper, we outline the implications of our findings for future research and higher education development.

## Theoretical background and state of research

### The Eccles et al. expectancy-value model

The expectancy-value model developed by Eccles and colleagues [15–18,20] was designed to identify the major categories of social and psychological influences on individuals’ achievement-related choices. It remains one of the most prominent theories in the field of motivation [21]. In this model, motivational variables such as *expectancies* for future (learning) success and *values* attributed to a given task are assumed to directly influence educational choices. According to Eccles et al., subjective task value may be extrinsic (utility value; e.g., usefulness for future career) or intrinsic (intrinsic value; e.g., interest in the subject). Value attribution also depends on perceived costs, such as the expected effort associated with a particular educational option (effort), fear of failure, or fear of disconfirming a salient personal or collective identity (psychological costs of failure).

The primary objective of this psychological expectancy-value model is to explain the genesis of values and expectancies through various (social) psychological mediating variables, such as ability beliefs, gender role stereotypes, and control beliefs. Among these factors, ability beliefs are particularly important, representing individuals’ subjective perceptions of their abilities in relation to specific domains, activities or educational options. Consequently, higher ability beliefs correlate with greater expectations of success in educational choices [22]. Additionally, ability beliefs can also influence individuals’ levels of interest and motivation [16–17]. Comparisons of domains within individuals were found to be critical for career choices. Individuals are likely to engage in activities where they feel most confident and that they value most ([22], pp. 10-11).

Ability beliefs themselves are shaped by individuals’ interpretations of their previous achievement-related experiences, such as grades, as well as by various socialisation influences, including gender, parental education, and migration background.

Applying this model to the transition from undergraduate to postgraduate studies, it can be expected that students will be inclined to pursue a master’s degree if they expect a high probability of academic success and significant career benefits, if they have a strong interest in the content of a master’s programme, and if they expect low levels of effort and psychological cost. Specifically, expectations of success, interest, and perceived effort are likely to depend on students’ evaluations of their previous academic achievement.

According to Eccles and colleagues, ability beliefs and interests need to be considered on a domain-specific basis [15–17,22]. In our context, this means supplementing ability beliefs related to a specific field of study with an assessment of ability beliefs related to aspects unique to postgraduate studies that are relevant across disciplines. Given the academic expectations associated with master’s degrees (in Germany, e.g., [23]), scientific work can be defined as such a distinct “domain” in postgraduate studies that is relevant across disciplines. In this domain, students acquire specific achievement-related experiences during their bachelor’s programme that can either encourage or discourage them from pursuing a scientific career, thereby influencing their decision-making regarding postgraduate studies [24]. Therefore, both students’ ability beliefs related to scientific work and their interest in engaging in scientific work are expected to play a significant role in the transition from undergraduate to postgraduate studies.

### State of research

The model developed by Eccles has been widely used to explain educational, vocational, and other achievement-related decisions, including course selection [25–27], choice of school track [28–30], and career choices [29–31]. Findings from these studies indicate that intrinsic value, expectancy of success, and ability beliefs are important predictors of educational decisions beyond grades and utility value [25,26,28,31]. However, as will be shown in the following section, these aspects have often been neglected in previous research on the transition from undergraduate to postgraduate studies, which has predominantly referred to sociological rational choice models that emphasise social inequality (e.g., [13–14]). To date, the Eccles et al model has not been empirically tested in the context of this transition.

#### Decision-making at the undergraduate to postgraduate transition.

Research on the transition from undergraduate to postgraduate education has mainly focused on inequalities in access to postgraduate education. Several studies have revealed that the decision to pursue postgraduate education is influenced not only by achievement-related characteristics, such as grades, but also by sociodemographic factors [3–5,9–12,32–34]. Since these inequalities have been extensively studied and are not the primary focus of the present study, we will concentrate in the following sections on presenting evidence on the explanatory power of individual components of the Eccles et al. model derived from these and other studies.

With regard to the importance of *expectations of success* in the transition from undergraduate to postgraduate education, only a limited number of studies are available. In most cases, the expectancy component has been measured indirectly, using variables such as grades or self-assessments of academic performance (e.g., [11,34]). However, Bergann and colleagues [24] have shown that a more direct measure of expectations of success is associated with transition intentions beyond mere grades and self-assessed performance (see also [10]). Their findings suggest that expectations of success are the strongest predictor of undergraduate students’ intentions to enter a master’s programme. The objective of the present study is to examine whether expectations of success retain this strong predictive power for the undergraduate-to-postgraduate transition when considering other value components.

Several studies from Europe and the United States have highlighted the influence of the different *value components*. For example, based on a German panel study, researchers found that anticipated instrumental returns (utility value) are linked to the decision or intention to pursue a master’s degree [10,34]. The results indicate that students are more likely to continue with a master’s degree, or express the intention to do so, if they associate the degree with high income, prestige, and favourable job prospects. The influence of these utility values has also been supported by other studies [2,4,24].

In addition to utility value, intrinsic value appears to play a significant role in the transition to a master’s programme. A German panel study revealed that high school graduates who prefer scientific activities over practical ones are more inclined to pursue postgraduate education later on [10,34]. Furthermore, De Boer and colleagues [2] found that the majority of students in the Netherlands cited intrinsic motives (e.g., acquisition of knowledge, interest in the subject) as reasons for choosing a master’s programme. These findings suggest that a strong interest in academic work is important for continuing on to postgraduate studies. However, the role of intrinsic value in this transition, especially when compared to utility value, has not been sufficiently investigated.

The influence of costs, as outlined in the Eccles et al. model (e.g., effort, psychological costs of failure), has not yet been examined in the context of the undergraduate-to-postgraduate transition. Previous studies have primarily concentrated on monetary cost aspects, such as income, debt, financial burden, or the prospect of early financial independence [4,8–11,24,34]. Collectively, these studies suggest that financial considerations do influence the decision to pursue a master’s degree, although they tend to play a minor role, particularly in countries like Germany, where tuition fees are low.

In summary, there is limited understanding of the relevance of the value and expectancy components specified in the Eccles et al. model for the undergraduate-to-postgraduate transition. Specifically, research on the influence of expectations of success and intrinsic value is lacking. Moreover, the relative importance of intrinsic value compared to utility value in this transition remains unclear. Investigating this issue would improve our understanding of whether students choose to remain in academia for a master’s degree because of a genuine interest in scientific work or simply because they associate a master’s degree with better career prospects. Furthermore, there is a notable lack of studies investigating the impact of effort or psychological costs of failure in this educational transition. Do these cost considerations exert a similar or even greater influence than monetary costs?

Additionally, most of the studies referenced above have measured expectancy and value components only indirectly (e.g., through grades, income, or debt) rather than directly assessing undergraduate students’ subjective judgements.

#### Ability beliefs as predictors of value and expectancy components.

In the Eccles et al. model, domain-specific ability beliefs play a crucial role as determinants of the value and expectancy components [16–17]. Based on Eccles’ expectancy-value model, the relationship between prior achievement-related experiences, ability beliefs, and educational choices during childhood and adolescence has received considerable empirical attention [16,17,22,35,36]. Findings from these early educational stages indicate that ability beliefs significantly influence students’ achievement trajectories and educational choices beyond their actual performance. These effects are partially mediated by the value and expectancy components (for a summary, see [16]). In addition, research has shown that ability beliefs are more strongly related to interest (intrinsic value) than to perceived usefulness (utility value) across domains [16,17]. In contrast, there is a notable lack of research examining the relationship between ability beliefs and expectations of success. The primary reason for this gap is that ability beliefs and expectations of success - two conceptually distinct constructs - were empirically highly correlated in childhood and adolescence, making it difficult to separate them by factor analysis [16,22]. Consequently, the two terms are often used interchangeably in the research literature [16,17,35]. However, longitudinal studies conducted during adolescence have revealed that ability beliefs in mathematics are related to both subsequent intrinsic values and expectations of success [16].

Regarding the transition from undergraduate to postgraduate studies, few studies have examined the relationship between ability beliefs and transition intentions, as well as possible mediating effects via the value and expectancy components. Some studies indicate that ability beliefs influence transition intentions beyond mere grades [10,24]. In line with the Eccles at al. model, Bergann and colleagues [24] found that the effect of ability beliefs on the intention to pursue a master’s degree is mediated by expectations of success. In this context, in addition to general ability beliefs in a particular field of study, students’ specific ability beliefs in relation to scientific work - namely their confidence in understanding and producing scientific texts - were also significant predictors of their expectations of success regarding a master’s degree programme. However, it remains unclear whether undergraduate students’ ability beliefs also influence their intrinsic values at the transition to postgraduate study, as posited by the Eccles et al. model.

## Research objectives

The objective of this study is to investigate which factors - beyond grades and sociodemographic and educational background characteristics - influence an individual’s decision to enrol in a master’s programme, based on the Eccles et al. expectancy-value model [15–17].

**Fig 1:**
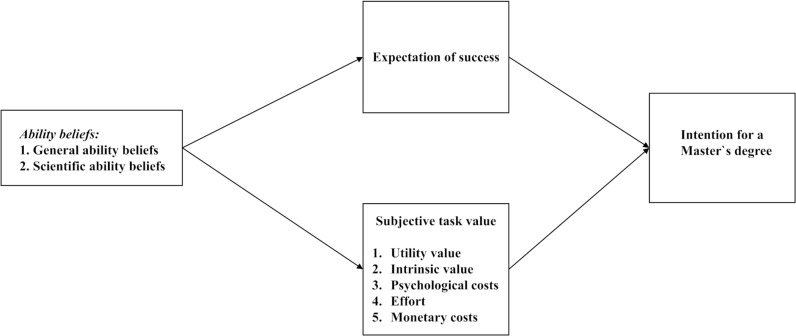
Simplified theoretical model for predicting intention for a master’s degree.

Fig 1 presents a simplified theoretical model. The dependent variable is the intention to pursue a master’s degree. The independent variables are the individual expectations of success and the value components (utility value, intrinsic value, expected effort, psychological costs of failure, and monetary costs). General and scientific ability beliefs are predictors of the expectancy and value components. The model is used to address the following research questions.

Our *first research question* is: What significance do the various expectancy and value components specified in Eccles’ model have for the intention to pursue a master’s degree? In addition to previous studies examining the decision-making processes at the transition from undergraduate to postgraduate education, we investigate the importance of intrinsic value, effort, and psychological costs in shaping students’ intentions to pursue a master’s degree, beyond the influence of utility value and monetary costs. Our first hypothesis is:

H1a: Intrinsic value, effort, and the anticipated psychological costs associated with a master’s degree contribute incrementally to explaining the intention to enrol in a master’s programme, even when controlling for expectations of success, utility value, and anticipated monetary costs.

Building on the findings of Bergann and colleagues [24], we also assume that expectations of success are most strongly associated with the intention to pursue a master’s programme. Therefore, this study proposes the following hypothesis:

H1b: The strongest predictor of the intention to pursue a master’s degree is expectation of success.

Our *second research question* is: In the transition from undergraduate to postgraduate education, what role do ability beliefs play in predicting the value and expectancy components? Ability beliefs have previously been identified as significant predictors of expectations of success, with a mediating effect on the intention to transition [24]. The present study is the first to examine the extent to which this relationship also applies to the value components as outlined in the Eccles et al. model. Consequently, we postulate the following hypothesis:

H2: The effect of ability beliefs on the intention to transition is mediated by both the expectancy component (expectations of success) and the value components (intrinsic value and effort).

## Method

The study is based on data collected from a cross-sectional online survey of undergraduate students of a major German university. The survey took place from June, 4th to July, 3th 2019. All students enrolled in a bachelor’s degree programme were invited by email to participate in the survey. Participation was voluntary, and the survey was conducted anonymously, as the project team did not have access to any personal data from participants. Consequently, informed consent was not necessary. As the survey is part of the university’s quality management system, all procedures were reviewed by the university’s data protection officer. Ethical approval was not required for this study.

The primary objective of the survey was to evaluate the university’s degree programmes. In addition, the survey included questions about the transition to postgraduate studies and gathered information on sociodemographic and educational background characteristics. As an incentive, participants could win an iPad, three Tolino e-book readers, and 45 vouchers worth 800 euros in total. Following two reminders, the survey achieved a response rate of 23 per cent.

### Sample

Responses were obtained from *N* =  3,044 undergraduate students (64% female) enrolled in 61 non-teaching degree programmes. 27 per cent of participants were studying natural sciences (e.g., biology, physics), 36 per cent were in social sciences (e.g., economics, psychology), and 37 per cent were pursuing humanities or cultural studies (e.g., philosophy, [art] history). The sample was broadly representative in terms of gender and subject groups. On average, students were *M* =  23.69 years old (*SD* =  5.36) and had completed *M* =  5.24 semesters at the time of the survey (*SD* =  2.97).

### Survey instruments

#### Expectancy-value model components.

Our operationalisation of the variables is shown in Table 1, which includes sample items and scale reliability information (Cronbach’s Alpha) for each variable. All scales demonstrated satisfactory to very good reliabilities. Both intentions for a master’s degree and expectations of success regarding the completion of a master’s programme were measured using two self-developed items each. Intrinsic value was operationalised by five items measuring students’ scientific interest, derived from a vocational interest questionnaire based on Holland [37]. Furthermore, four items measuring utility value (e.g., high income, good career prospects) and a single item assessing anticipated monetary costs were sourced from Lörz and colleagues [38]. Psychological costs of failure were measured using a single item from a questionnaire by Hagemeier and Murwaski [39]. Since this item measures the psychological costs associated with *not* obtaining a master’s degree, it was recoded so that lower values indicated lower costs. Effort was measured using a self-developed item. All items, except those measuring scientific interest, used six-point response scales.

**Table 1:** Operationalisation of expectancy-value model components and scale reliabilities.

Construct (number of items)	Sample item	Cronbach’s Alpha
Intention to pursue a master’s degree (2 items)^1^	*“After completing my bachelor’s degree, I want to pursue a master’s degree.”*	.94
Expectations of success (2 items)^1^	*“I am confident that I can successfully complete a master’s degree.”*	.86
Intrinsic value (scientific interest; 5 items)^2^	*“Please rate the following activities in terms of how much they interest you or might interest you: investigating the roots of a problem”*	.81
Utility value (4 items)^3^	*“To what extent do you associate the following aspects with a master’s degree: safe job”*	.89
Monetary costs (single item)^3^	*“To what extent do you associate the following aspects with a master’s degree: high financial burden”*	–
Psychological costs of failure (single item)^1^	*“My family would be disappointed if I didn’t earn a master’s degree.”* (recoded)	–
Effort (single item)^1^	*“I would have to invest a lot of time and effort to earn a good master’s degree.”*	–
General ability beliefs (2 items)^1^	*“Compared to my fellow students, I earned better grades in my university studies.”*	.65
Scientific ability beliefs (3 items)^1^	*“I often find it difficult to understand scientific texts.”* (recoded)	.79

*Note.* Response scales:

^1^1 = *completely disagree* to 6 = *fully agree*,

^2^1 = *not at all* to 5 = *very* *much*,

^3^1 = *not at all* to 6 = *very much.*

The two items assessing general ability beliefs in the field of study were taken from Köller and colleagues [40]. To measure scientific ability beliefs, three items from Bergann et al. [24] were used, focusing on the ability to understand scientific texts and to express oneself in a scientific way. We singled out the aspects of understanding and producing scientific texts, because these skills are relevant across all master’s degree programmes. Moreover, these abilities can be assessed by bachelor’s degree students from the beginning of their studies, which is important since we aimed to survey students from all semesters. Four items with a negative wording were recoded so that higher values indicate higher levels of the respective constructs.

#### Covariates.

Grades, along with sociodemographic and educational background characteristics, were included as covariates in our models, as these factors have been shown to be significant in explaining the transition from undergraduate to postgraduate education. Two grades were considered in our analyses: the grade of the university entrance qualification and the average grade of students’ undergraduate academic work. Grades were recoded for the analysis so that high scores represent good grades and low scores indicate poor grades. Additionally, the current undergraduate average grade was z-standardised within subjects to reflect relative differences in achievement, independent of variations in grading practices across disciplines. To capture students’ gender (0 =  female, 1 =  male), parental education level (0 =  no university background, 1 =  at least one parent holds a university degree), and migration background (0 =  no migration background, 1 =  students and/or both parents were born abroad), three dummy variables were created. Furthermore, students’ age was included in the analyses. Additional educational covariates comprised the completion of vocational training prior to commencing the bachelor’s degree (0 =  no, 1 =  yes) and the extent to which students worked alongside their studies (measured in average weekly working hours). We also categorised students’ fields of study into three broad groups: social sciences, humanities and cultural studies, and natural sciences. To account for the potential impact of students’ current stage in their programme on their intention to pursue a master’s degree, we included a variable that classified students into three groups: first- and second-year students ( < 5 semesters), students nearing the end of the normal programme duration (5-8 semesters), and students who had exceeded the normal programme length ( > 8 semesters).

### Statistical and analytical procedure

To address our research questions, we computed a structural equation model. This approach enabled us to simultaneously test our hypotheses regarding the correlations between the expectancy and value components and the intention to pursue a master’s degree, including potential mediating effects. As shown in Fig 1, the intention to pursue a master’s degree is the dependent variable, and the expectancy and value components were integrated into the model as predictors of this intention, along with ability beliefs. The model also includes mediation effects to explore whether the impact of ability beliefs on the intention to pursue a master’s degree is mediated by the expectancy and value components. Components measured with multiple items were modelled as latent variables. For clarity, a simplified representation of the structural equation model is provided in the results section. A presentation of the measurement models as well as the residual covariances in the structural model has been omitted. In line with our theoretical considerations, grades and sociodemographic and educational background covariates were included in the model as predictors of the expectancy-value model components. The threshold for significance was *p* < .05. The full results of the regression analyses conducted are provided in the S1 Appendix, which includes all estimated regression coefficients, their standard errors, p-values, and confidence intervals.

All analyses were performed using the statistical software Mplus (version 8.1; [41]). Maximum likelihood estimation with robust standard errors (MLR estimator) was used to account for possible non-normality in the measures. The calculation of standard errors, takes into account the multilevel structure of the data, i.e., the grouping of students into *N* =  61 subjects (cluster variable) using the option *type =  complex*. Missing values were present in the dependent and independent variables, as well as in the covariates, ranging from 0 per cent for the number of semesters to 14 per cent for the average undergraduate grade. On average, 8 per cent of the values were missing. We therefore used the Full Information Maximum Likelihood (FIML) method implemented in Mplus to avoid bias in parameter estimation and a reduction in test power [42]. To examine the significance of the mediation effects, we used 95% bootstrapping confidence intervals with 2000 iterations. BCBOOTSTRAP option was used to get bias-corrected confidence intervals [43]. Mediation effects were assumed to be significant if the confidence interval did not include the value zero.

## Results

### Descriptive results

The intention to pursue a master’s degree was notably high in the sample, with a mean score of *M* =  4.71 (*SD* =  1.59) on a six-point response scale (see Table 2). All bivariate correlations were in the anticipated direction: expectations of success (*r* = .54, *p* < .001), the anticipated instrumental returns of a master’s degree (utility value: *r* = .29, *p* < .001), and scientific interest (intrinsic value: *r* = .34, *p* < .001) were positively associated with the intention to pursue a master’s degree. Conversely, anticipated costs (psychological costs of failure: *r* = -.17, *p* < .001; monetary costs: *r* =  -.08, *p* < .001) as well as the perceived effort required to successfully complete a master’s degree (effort: *r* =  -.10, *p* < .001) were negatively associated with this intention.

**Table 2:** Means (Standard deviations) for all model variables and bivariate correlations with the dependent variable (Intention to pursue a master’s degree).

Variable	*M (SD)*	Bivariate correlation	p-Value
Intention to pursue a master’s degree	4.71 (1.59)	–	–
*Expectancy-value model components*			
General ability beliefs	3.50 (1.27)	.26***	>.001
Scientific ability beliefs	4.27 (1.11)	.25***	>.001
Expectations of success	4.81 (1.22)	.54***	>.001
Utility value	4.15 (1.36)	.29***	>.001
Intrinsic value	3.93 (0.73)	.34***	>.001
Psychological costs of failure	4.68 (1.72)	-.17***	>.001
Monetary costs	3.15 (1.51)	-.08***	>.001
Effort	4.64 (1.30)	-.10***	>.001
*Covariates*			
Grade in the university entrance qualification (recoded)	3.00 (0.62)	.21***	>.001
Grade in the undergraduate programme (recoded & standardised)	-0.01 (0.53)	.22***	>.001
Gender (Ref.: female)	0.36 (0.48)	-.00	.975
Parental education level (Ref.: no university background)	0.59 (0.49)	.11**	.001
Migration background (Ref.: no)	0.35 (0.48)	-.02	.469
Age	23.69 (5.36)	-.16***	>.001
Completed vocational training (Ref.: no)	0.15 (0.36)	-.17***	>.001
Average weekly working hours	9.28 (8.74)	-.10***	>.001
Natural sciences (Ref.)	–	–	
Social sciences	0.36 (0.48)	.03	.728
Humanities and cultural studies	0.37 (0.48)	-.12	.082
Number of semesters: First- and second year students (Ref.)	–	–	–
Number of semesters: End of normal programme length	0.39 (0.49)	.04	.063
Number of semesters: Exceeding normal programme length	0.09 (0.29)	-.21***	>.001

*Note. N* =  3,044. Fully standardised Pearson’s bivariate correlation coefficients

*p < .05, **p < .01, ***p < .001.

Furthermore, both general and scientific ability beliefs (general: *r* = .26, *p* < .001/scientific: *r* = .25, *p* < .001), as well as grades (university entrance qualification: *r* = .21, *p* < .001/undergraduate: *r* = .22, *p* < .001) were positively associated with the intention to pursue a master’s degree.

The bivariate correlations between the intention to pursue a master’s degree and sociodemographic and educational background variables were largely consistent with existing literature (see Table 2). Specifically, parental education level showed a positive correlation with the intention to pursue a master’s degree (*r* = .11, *p* = .001), while age (*r* =  -.16, *p* < .001), average weekly working hours (*r* =  -.10, p < .001), and completion of vocational training (*r* =  -.17, *p* < .001) were negatively associated with this intention. Notably, students who had (significantly) exceeded the normal programme length were less likely to intend to pursue a master’s degree (*r* =  -.21, p < .001), while students at the end of the normal programme duration did not exhibit a higher intention to start a master’s degree compared to first- and second-year students.

### Predictors of the intention to pursue a master’s degree

**Fig 2:**
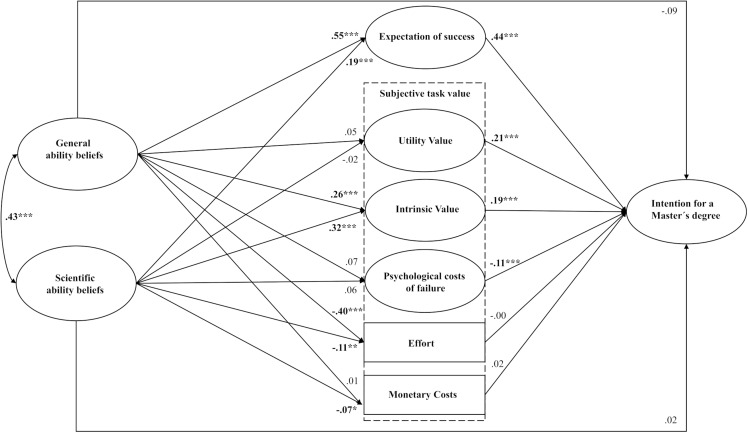
Structural equation model predicting the intention to pursue a master’s degree (simplified representation). *Note:* Fully standardised coefficients; Latent variables are shown as ellipses; ***p < .001, **p < .01, *p < .05.

Fig 2 shows a simplified representation of the structural equation model predicting the intention to pursue a master’s degree. Model fit is excellent (χ2 (293) =  1093.40, *p* = .000, *RMSEA* = .030, *CFI* =  0.967, *TLI* =  0.948, *SRMR* = .023).

The right side of Fig 2 illustrates the relationships between the expectancy and value components and students’ intentions to pursue a master’s degree. Both expectations of success (*β* = .44, *p* < .001) and the utility value and intrinsic value components had a significant positive effect on the intention to pursue a master’s degree (*β* = .21, *p* < .001 and *β* = .19, *p* < .001, respectively). As expected, higher expectations of success and greater assessments of both the extrinsic and the intrinsic value of a master’s degree were associated with a stronger intention to enrol. Using model constraint statements, we tested whether the standardised regression coefficients for the two value components (utility value and intrinsic value) differed significantly. A comparison of the coefficients revealed that both aspects exerted a similarly strong influence on the intention to pursue a master’s degree (*p* = .721). Contrary to our expectations, monetary costs and perceived effort did not demonstrate significant effects. Consistent with our expectations, psychological costs of failure had a significant negative effect on students’ intentions to pursue a master’s degree, even when controlling for the other model variables (*β* =  -.11, *p* < .001). Students’ transition intentions were higher when they anticipated disappointing their parents by not engaging in postgraduate studies. Therefore, as expected in H1a, intrinsic value and psychological costs of pursuing a master’s degree contribute incrementally to explaining the intention to enrol, even when controlling for expectations of success, utility value, and anticipated monetary costs. Expectations of success emerged as the strongest predictor of the intention to pursue a master’s degree, as expected in H1b.

Moreover, the effects of ability beliefs on the intention to pursue a master’s degree were found to be fully mediated by the expectancy and value components (Fig 2), with no significant direct effects observed. In total, 42 per cent of the variance in the dependent variable was explained by the model variables and covariates.

Turning to the left side of Fig 2, we observe that both general subject-related and scientific ability beliefs were positively associated with expectations of success (*β* = .55, *p* < .001 and *β* = .19, *p* < .001, respectively) and scientific interest (intrinsic value: *β* = .26, *p* < .001 and *β* = .32, *p* < .001, respectively), while being negatively associated with perceived effort (*β* =  -.40, *p* < .001 and *β* =  -.11, *p* = .001, respectively). Furthermore, scientific ability beliefs were negatively correlated with anticipated monetary costs (*β* =  -.07, *p* = .021). Students who rated their scientific ability higher believed they would incur lower monetary costs from a master’s degree.

We conducted mediation analyses to examine the extent to which the effects of ability beliefs were mediated through the expectancy and value components. No mediation effects were identified for the value components of effort, monetary costs, psychological costs, and utility value. The results indicated that the effect of general ability beliefs on the intention to pursue a master’s degree was mediated by both expectations of success and scientific interest (intrinsic value). The mediation effect for scientific interest (*β* = .05, *SE* =  0.01*,* 95% CI [0.03, 0.08]) was approximately one-fifth of that for expectations of success (*β* = .25, *SE* =  0.05*,* 95% CI [0.15, 0.34]). Similarly, the effect of scientific ability beliefs on intention to pursue a master’s degree was mediated by both expectations of success and scientific interest, with the mediation effect for intrinsic value (*β* = .06, *SE* =  0.01*,* 95% CI [0.04, 0.09]) being slightly smaller, but corresponding to about three-quarters of the mediation effect for expectations of success (*β* = .08, *SE* =  0.02, 95% CI [0.04, 0.12]) in this context.

Thus, as expected in H2 and in line with the Eccles et al. model, the effects of ability beliefs on intention to pursue a master’s degree were mediated by both the expectancy and value components, with intrinsic value acting as a particularly important mediator. This suggests that students with higher ratings of their general and scientific abilities had higher transition intentions due to their higher scientific interest and higher expectations of success.

Table 3 presents the standardised regression coefficients between grades and sociodemographic and educational background characteristics, alongside the expectancy-value model components. The bivariate correlations between intention to pursue a master’s degree and the covariates can be largely attributed to differences in the expectancy-value model components. However, students with lower high school grades and those who engaged more in part-time employment during their studies had a somewhat lower intention to pursue a master’s degree, even after controlling for model variables. The same was true for students who had significantly exceeded the normal programme length. Furthermore, as expected, significant correlations were identified between the covariates and the ability beliefs, as well as the value and expectancy components (see Table 3). Extrinsic and intrinsic motives for the transition from undergraduate to postgraduate education varied not only based on students’ sociodemographic characteristics but also, notably, on the subject group of their undergraduate programme.

**Table 3:** Regressions of model variables on grades and sociodemographic and educational background characteristics (fully standardised regression coefficients).

	General ability beliefs	Scientific ability beliefs	Expectations of success	Utility value	Intrinsic value	Psycholo-gical costs	Effort	Monetary costs	Intention to pursue a master’s degree
*Grades*									
Grade in the university entrance qualification	.09**	.12**	.02	-.01	.05	-.12***	-.01	-.07***	.05*
Grade in undergraduate programme	.76***	.28***	-.16*^a^	-.07	-.10* ^a^	-.06	.13* ^a^	-.03	.06
*Sociodemographic and educational background characteristics*									
Gender (Ref.: female)	.06**	.14***	-.01	-.01	.01	-.01	.06***	-.03	-.02
Parental education level (Ref.: no university background)	-.00	.07***	.04	-.01	-.00	-.16***	-.03	-.01	-.01
Migration background (Ref.: no)	-.03	-.12***	.00	.07***	-.00	-.14***	.03	.03	-.01
Age	.03	.09**	.00	-.08*	.13***	.09***	.01	.05	-.05
Completed vocational training (Ref.: no)	.01	-.02	-.04	-.00	-.00	.04*	.05*	.02	-.03
Average weekly working hours	.00	.06**	-.02	-.04*	.03	.03	-.06**	.06*	-.05*
Social sciences (Ref.: natural sciences)	.07	-.02	-.02	.03	-.17*	-.05	-.10***	.01	-.05
Humanities and cultural studies (Ref.: natural sciences)	.19***	.04	-.13***	-.29***	-.09*	-.03	**-.**05	.06*	-.02
Number of semesters: end of normal programme length (Ref.: < 4 semesters)	.00	.05	.05	-.04	-.06*	-.08***	-.03*	-.03	-.01
Number of semesters: exceeding normal programme length (Ref.: < 4 semesters)	-.03	.03	.01	-.03	-.02	-.07**	-.01	.04	-.08**
R^2^	.659	.169	.332	.115	.247	.108	.172	.042	.417

*Note*. ^a^ These effects are not interpreted in terms of content, because they are suppressor effects due to the multicollinearity between the variables “grade in undergraduate programme” and “general ability beliefs”.

***p < .001, **p < .01, * p < .05.

## Summary and discussion

This paper is the first to apply key components of the Eccles et al. expectancy-value model [15,16] to the transition from undergraduate to postgraduate studies. Most previous studies have used sociological rational choice models that focus primarily on probabilities of success (grades), instrumental values, and monetary costs to explain transition intentions [4,8,10,11,34]. However, Eccles’ psychological expectancy-value model describes additional value components, such as intrinsic motives and psychological costs, as well as determinants of the expectancy and value components. Our findings indicate that these motivational factors, which have been largely neglected in previous research, play an important role in students’ decisions during the transition from undergraduate to postgraduate education.

Understanding students’ motivations for entering postgraduate education is particularly important given the high rates of transition to master’s programmes at universities across Europe, which remain notable 20 years after the implementation of the Bologna reforms [5,7,8]. What role do performance-related considerations play in the transition from undergraduate to postgraduate studies, and what other motives influence students’ decisions? Do undergraduate students pursue a master’s programme primarily for extrinsic motives (e.g., good career opportunities, job security) rather than intrinsic motives (e.g., interest in scientific work)? This could suggest that the bachelor’s degree is not recognised as a full professional qualification in Germany, leading students to believe that a master’s degree is necessary for a successful career.

Regarding our first research question on the role of the expectancy and value components, we found that students’ *expectations of success* were the strongest predictor of their intention to transition providing an incremental contribution to predicting intentions for a master’s degree beyond grades and ability beliefs (cf. [24]). In terms of value components, *intrinsic motives* (i.e., strong academic interest) were found to be as important as *extrinsic motives* (i.e., career and income considerations). Thus, our results do not suggest that it is primarily extrinsic motives that drive undergraduate students to enter postgraduate education. The assumption that high transition rates to master’s programmes are primarily driven by students’ belief in the necessity of a master’s degree for professional success cannot be confirmed. Instead, intrinsic motivation plays a significant role, independent of and in addition to achievement-related considerations (cf. [2,10,34]). Given that the intrinsic value component was operationalised only indirectly via scientific interest in the present study, its effect may even have been underestimated. Other intrinsic motives, such as interest in the subject, which were not measured in this study, are also likely to play a role. Thus, future research should place more emphasis on intrinsic motives during this transition.

Another finding from our study is that cost considerations were of minor importance for the intention to enrol in a master’s programme. No association was found between anticipated *monetary costs* and transition intention after controlling for other model variables. Similar results have also been reported in other European studies [8,10,24]. However, operationalising monetary costs through a single item (anticipated financial burden) does not allow for general conclusions about the effect of this characteristic. Other aspects of costs (e.g., income, opportunity cost) that may influence transition behaviour as well [4,9–11] were not considered in our study. Furthermore, *expected effort* did not demonstrate a significant correlation with the intention to pursue a master’s degree after controlling for grades and ability beliefs. In contrast, *psychological costs* had a significant effect, as postulated by the theoretical model [15–17]. Students who feared disappointing their families by not obtaining a master’s degree had higher transition intentions, even after controlling for academic achievement. In light of these results, future studies should also consider additional cost components beyond the already well-explored monetary costs.

The second research question addressed the role of ability beliefs as factors conditioning the expectancy and value components during the transition from undergraduate to postgraduate education. Our study demonstrated that study-related ability beliefs are associated with both expectations of success and scientific interest, and that the effects of these ability beliefs on transition intentions are mediated by expectations of success and scientific interest. Consistent with prior research [22,24], we found that considering both general subject-related and specific scientific ability beliefs was valuable. Scientific ability beliefs, in addition to grades and general subject-related ability beliefs, significantly contributed to predicting transition intentions. Undergraduate students who rated their scientific abilities lower had lower expectations of success, lower scientific interest, and were less likely to intend to pursue a master’s degree, even after controlling for academic performance. In accordance with the theoretical model, both general and scientific ability beliefs were influenced by both achievement-related factors, such as grades, and non-achievement-related factors, such as gender, parental education level and migration background.

Regarding the timing of students’ decision to pursue a master’s degree, we found that this decision is made early in their academic career. Whether students were at the beginning of the undergraduate programme or nearing completion did not affect their intention to pursue a master’s degree. This finding is further supported by the significant influence of the grade in the university entrance qualification on the intention to start a master’s programme, even after controlling for other model variables.

In summary, although the Eccles et al. model was primarily developed to explain horizontal educational inequalities, our study confirms its value for explaining individual choices during the transition from undergraduate to postgraduate education. It highlights the importance of factors that have largely been neglected in previous research, such as expectations of success, intrinsic motives, and psychological costs.

## Limitations

The following factors limit the generalisation of our findings. First, due to our cross-sectional design, causal inferences cannot be drawn. Moreover, our survey captured only students’ intentions to pursue a master’s programme and not their actual transition. Longitudinal studies are necessary to validate the findings. Another limitation is that our data refer to a single institution, which may limit the generalisability of our findings. Future research should explore whether our results hold in other universities, and in different country contexts. An important consideration in this regard is the potential impact of financial factors, as it is reasonable to assume that financial considerations play a more significant role in countries where tuition fees are charged, such as the United States or the United Kingdom.

## Implications

In addition to advancing the theoretical understanding of the bachelor-to-master transition, our findings have some practical implications for universities. We found that the decision to pursue a master’s degree is often made early in students’ academic careers, with their assessments of scientific abilities and interest in scientific work playing crucial roles. For universities, this suggests that providing undergraduate research opportunities, even in the early stages of the bachelor’s programme, could facilitate well-informed postgraduate choices. Enhancing research-based teaching at the undergraduate level may stimulate students’ interest in research and improve their acquisition of research skills, thereby strengthening their expectations of successfully completing a master’s degree (e.g., [44]).

Our results indicate that extrinsic and intrinsic motives are of comparable importance in the decision to pursue postgraduate studies. However, it remains unclear whether individuals have multiple motives (intrinsic and extrinsic) for obtaining a master’s degree simultaneously, or if some individuals prioritise extrinsic motives while others are primarily driven by intrinsic motives. Future research could employ person-centred methods, such as Latent Profile Analysis, to gain deeper insights into the interplay between extrinsic and intrinsic motives. In any case, we found that the importance of extrinsic and intrinsic motives varies across fields of study. Extrinsic motives played a smaller role in the humanities and cultural studies, and intrinsic motives played a smaller role in the social sciences than in the natural sciences (see Table 3). This pattern of findings is likely to reflect the labour market dynamics within the respective fields. Programmes that qualify students for prestigious careers and promise good labour market prospects are more likely to attract students with career goals, whereas fields of study with less clear career prospects may be chosen more for pure interest in the subject and academic work. This insight can be used when designing and further developing degree programmes to create programmes that better align with the interests of students in each field.

## Supporting information

S1 Appendix Tables. (PDF)

S1 File Data Bergann et al-master.xlsx.
(XLSX)
