# Supplementary material for: Intrinsic and extrinsic motives of undergraduate students for pursuing a master’s degree: Applying the Eccles et al. expectancy-value model
Source: PLoS One. 2025 Mar 25;20(3):e0317204. doi: 10.1371/journal.pone.0317204 (PMC11936261; doi:10.1371/journal.pone.0317204)
Supplement: S1 Appendix — (PDF) [file pone.0317204.s001.pdf]

S1 Table. Regression of Intention for a master`s degree on expectancy and value components, ability beliefs, grades, and sociodemographic and education background characteristics (fully standardized regression coefficients, 95% confidence intervals, standard errors and p-value)

	Intention for a master`s degree		
	β [CI]	S.E.	p
Expectation of success	**.44** [.41,.48]	.02	.000
Utility value	**.21** [.16,.25]	.02	.000
Intrinsic value	**.19** [.15,.23]	.02	.000
Psychological costs of failure	**-.11** [-.14,-.07]	.02	.000
Effort	-.00 [-.05,.04]	.02	.859
Monetary Costs	.02 [-.02,.06]	.02	.303
General ability beliefs	-.09 [-.20,.02]	.06	.090
Scientific ability beliefs	.02 [-.04,.07]	.03	.507
Grade in the university entrance qualification	**.05** [.01,.09]	.02	.010
Grade in undergraduate programme	.06 [-.03,.14]	.04	.154
Gender (Ref.: female)	-.02 [-.06,.01]	.02	.224
Parental education level (Ref.: no university background)	-.01 [-.05,.02]	.02	.478
Migration background (Ref.: no)	-.01 [-.05,.02]	.02	.506
Age	-.05 [-.11,.02]	.03	.156
Completed vocational training (Ref.: no)	-.03 [-.08,.01]	.02	.149
Average weekly working hours	**-.05** [-.09,-.01]	.02	.028
Social sciences (Ref.: natural sciences)	-.05 [-.12,.03]	.04	.268
Humanities and cultural studies (Ref.: natural sciences)	-.02 [-.11,.07]	.05	.701
Number of semesters: end of normal programme length (Ref.: <4 semesters)	-.01 [-.04,.02]	.02	.452
Number of semesters: exceeding normal programme length (Ref.: <4 semesters)	**-.08** [-.12,-.03]	.02	.001
R^2		.417	

Note: β = fully standardized regression coefficients; CI = 95% confidence interval; S.E. = standard error; p = p-value

S2 Table. Regressions of expectancy and value components on ability beliefs, grades and sociodemographic and education background characteristics (fully standardized regression coefficients)

	Expectation of success			Utility value			Intrinsic value			Psychological costs			Effort			Monetary costs		
	β [CI]	S.E.	p	β [CI]	S.E.	p	β [CI]	S.E.	p	β [CI]	S.E.	p	β [CI]	S.E.	p	β [CI]	S.E.	p
General ability beliefs	**.55** [.36,.72]	.09	.000	.05 [-.07,.18]	.06	.406	**.26** [.15,.37]	.06	.000	.07 [-.07,.21]	.07	.319	**-.40** [-.55,-.27]	.07	.000	.01 [-.07,.13]	.05	.808
Scientific ability beliefs	**.19** [.10,.27]	.04	.000	-.02 [-.09,.04]	.03	.484	**.32** [.26,.38]	.03	.000	.06 [-.00,.12]	.03	.053	**-.11** [-.18,-.03]	.04	.004	**-.07** [-.14,-.03]	.03	.021
Grade in the university entrance qualification	.02 [-.03,.07]	.03	.363	-.01 [-.07,.05]	.03	.765	.05 [-.01,.11]	.03	.083	**-.12** [-.17,-.18]	.02	.000	-.01 [-.06,.04]	.03	.637	**-.07** [-.11,-.03]	.02	.000
Grade in undergraduate programme	**-.16** [-.25,-.00]	.07	.019	-.07 [-.17,.03]	.05	.157	**-.10** [-.19,-.01]	.05	.030	-.06 [-.15,.05]	.05	.247	**.13** [.04,.25]	.05	.013	-.03 [-.12,.02]	.04	.399
Gender (Ref.: female)	-.01 [-.04,.03]	.02	.756	-.01 [-.06,.03]	.02	.540	.01 [-.03,.06]	.02	.569	-.01 [-.03,.02]	.01	.701	**.06** [.03,.09]	.02	.000	-.03 [-.08,.02]	.03	.234
Parental education level (Ref.: no university background)	.04 [-.00,.07]	.02	.078	-.01 [-.05,.04]	.02	.704	-.00 [-.05,.04]	.02	.876	**-.16** [-.19,-.12]	.02	.000	-.03 [-.06,.00]	.02	.166	-.01 [-.06,.03]	.02	.585
Migration background (Ref.: no)	.00 [-.04,.04]	.02	.982	**.07** [.03,.11]	.02	.000	-.00 [-.05,.04]	.02	.897	**-.14** [-.18,-.11]	.02	.000	.03 [-.01,.07]	.02	.127	.03 [-.01,.06]	.02	.117
Age	.00 [-.04,.05]	.02	.865	**-.08** [-.14,-.01]	.03	.020	**.13** [.09,.18]	.03	.000	**.09** [.06,.13]	.02	.000	.01 [-.05,.06]	.03	.798	.05 [-.01,.12]	.03	.124
Completed vocational training (Ref.: no)	-.04 [-.08,.01]	.03	.145	-.00 [-.06,.05]	.03	.979	-.00 [-.05,.05]	.03	.931	**.04** [.01,.07]	.02	.023	**.05** [.00,.09]	.02	.044	.02 [-.03,.07]	.03	.386
Average weekly working hours	-.02 [-.07,.03]	.03	.502	**-.04** [-.08,-.00]	.02	.044	.03 [-.02,.07]	.03	.251	.03 [.00,.07]	.02	.097	**-.06** [-.10,-.03]	.02	.001	**.06** [.01,.10]	.02	.020

Social sciences (Ref.: natural sciences)	-.02 [-.07,.03]	.03	.371	.03 [-.09,.15]	.06	.630	**-.17** [-.31,-03]	.07	.016	-.05 [-.10,.01]	.03	.077	**-.10** [-.14,-.05]	.02	.000	.01 [-.06,.08]	.03	.757
Humanities and cultural studies (Ref.: natural sciences)	**-.13** [-.18,-.07]	.03	.000	**-.29** [-.37-.21]	.04	.000	**-.09** [-.19,-.00]	.05	.047	-.03 [-.07,.01]	.02	.179	-.05 [-.09,.01]	.03	.068	**.06** [.00,.11]	.03	.036
Number of semesters: end of normal programme length (Ref.: <4 semesters)	.05 [-.00,.09]	.02	.053	-.04 [-.08,.01]	.02	.108	**-.06** [-.11,-.02]	.03	.017	**-.08** [-.13,-.05]	.02	.000	**-.03** [-.06,.00]	.02	.045	-.03 [-.08,.02]	.02	.174
Number of semesters: exceeding normal programme length (Ref.: <4 semesters)	.01 [-.05,.06]	.03	.801	-.03 [-.07,.02]	.02	.227	-.02 [-.07,.01]	.02	.264	-.07 [-.11,-.03]	.02	.097	-.01 [-.05,.02]	.02	.499	.04 [-.01,.09]	.03	.092
R^2	.332			.115			.247			.108			.172			.042		

Note: β = fully standardized regression coefficients; CI = 95% confidence interval; S.E. = standard error; p = p-value

S3 Table. Regressions of ability beliefs on grades and sociodemographic and education background characteristics (fully standardized regression coefficients)

	General ability beliefs			Scientific ability beliefs		
	β [CI]	S.E.	p	β [CI]	S.E.	p
Grade in the university entrance qualification	**.09** [.04,.15]	.03	.001	**.12** [.05,.19]	.03	.001
Grade in undergraduate programme	**.76** [.73,.79]	.02	.000	**.28** [.22,.32]	.03	.000
Gender (Ref.: female)	**.06** [.02,.10]	.02	.002	**.14** [.11,.18]	.02	.000
Parental education level (Ref.: no university background)	-.00 [-.04,.03]	.02	.943	**.07** [.04,.11]	.02	.000
Migration background (Ref.: no)	-.03 [-.07,.01]	.02	.110	**-.12** [-.16,-08]	.02	.000
Age	.03 [-.03,.08]	.03	.218	**.09** [.03,.15]	.03	.002
Completed vocational training (Ref.: no)	.01 [-.04,.06]	.03	.595	-.02 [-.08,.03]	.03	.440
Average weekly working hours	.00 [-.04,.04]	.02	.971	**.06** [.02,.10]	.02	.005
Social sciences (Ref.: natural sciences)	.07 [-.01,.16]	.04	.084	-.02 [-.10,.06]	.04	.590
Humanities and cultural studies (Ref.: natural sciences)	**.19** [.12,.26]	.04	.000	.04 [-.05,.12]	.04	.378
Number of semesters: end of normal programme length (Ref.: <4 semesters)	.00 [-.03,.04]	.02	.995	.05 [.00,.10]	.02	.057
Number of semesters: exceeding normal programme length (Ref.: <4 semesters)	-.03 [-.06,.01]	.02	.108	.03 [-.02,.08]	.03	.290
R^2		.659			.169	

Note: β = fully standardized regression coefficients; CI = 95% confidence interval; S.E. = standard error; p = p-value
